# Smoke dispersion test and emergency control plan of fire in mine roadway during downward ventilation

**DOI:** 10.1038/s41598-023-30779-6

**Published:** 2023-03-06

**Authors:** Dongjie Hu, Zongxiang Li, Haiwen Wang, Haoyu Xu, Chuntong Miao

**Affiliations:** 1 College of Safety Science and Engineering, Liaoning Technical University, Liaoning, 123000 China; 2 Key Laboratory of Mine Thermodynamic Disasters and Control of Ministry of Education, Liaoning Technical University, Liaoning, 123000 China; 3 Ansteel Group Mining Co. Ltd., Liaoning, 114000 China; 4 CCTEG Chongqing Research Institute, Chongqing, 400039 China; 5 China Coal Technology and Engineering Group Shenyang Research Institute, Fushun, 113122 China

**Keywords:** Natural hazards, Engineering

## Abstract

To explore the wind flow turbulence and smoke flow diffusion law during the mine downward ventilation fire, two similar experimental platforms of a inclined single pipe test device and a loop system multiple pipe test device were built. The change data of the air flow in the pipeline during the fire period under different air volumes were measured. The evolution process of downward ventilation fire in the whole roadway network domain in Dayan Mine was simulated, and the emergency plan was put forward. The results show that in the experiment, the combustion intensity of the fire source is positively correlated with the ventilation power, and the fire wind pressure increases with the increase of the inclination angle of the pipeline. The throttling effect of the fire area and the combustion of the fire source together make the air volume in the pipeline change rapidly. The critical wind speed that makes the downward ventilation flow fire wind pressure equal to the fan power is 1.8 m s^−1^. The stronger the fan capacity, the stronger the ability of the main air path to overcome the resistance of the fire zone and maintain the original state. In the simulation, the most dangerous place when the downward ventilation fire smoke is reversed is the area (weak flow area) in the mine tunnel network where the ventilation power is weaker than the fire wind power. This study can provide a theoretical basis for the formulation of emergency plans for mine fire accidents.

## Introduction

The high-temperature toxic smoke generated by the exogenous fire spreads in the mine, causing poisoning or suffocation. Downdraft ventilation fires are very specific and complex. Due to the existence of fire-heating air pressure, when a fire occurs in the tunnel with downward ventilation, the phenomenon of wind flow weakening, smoke flow reversal, and wind backflow may occur. So it is more harmful and more difficult to control. It is of great theoretical value and practical significance to explore the distribution law of wind flow disturbance, smoke flow diffusion and temperature during the mine downdraft ventilation fires period for the prevention and control of fires.

Scholars have made great progress in mine fire smoke diffusion and fire thermal dynamics^1–4^. In 1997, Jiang^5^ used the SIMPLE algorithm to numerically solve a model of high temperature smoke flow propagation and heat and mass transfer during mine fires and compared the results with the measured data to verify the accuracy of the established model the author also studied the influence laws of different fire source characteristics, tunnel properties, and ventilation conditions on the fire smoke flow, laying a foundation for future fire simulation studies. In 2004, Zhou^6^ developed a smoke flow rollback model and conducted numerical simulations to study the effects of the air supply and heat release rate conditions on the smoke flow rollback distance during mine fires using the computational fluid dynamics software PHOENICS, based on the conservation principle. In 2015, Li^7^ created a smoke diffusion model during underground fires by analyzing factors such as double diffusion effects, mine ventilation, buoyancy function, and throttle action. It describes the temporal and spatial development of fire intensity and smoke diffusion in underground fires. In 2016, Qi^8^ obtained the spreading pattern of beltway fire smoke flow in an accident-prone belt transport lane by numerical simulation. In 2016, Chen^9^ used FDS to perform a 3D numerical simulation of high-temperature smoke flow in a single lane during the fire period to divide the safety zone based on the degree of injury to people from high temperatures and CO concentration. In 2017, Fan^10^ used fire dynamics simulation software to simulate the fire development process in horizontal roadway, and studied the smoke movement under the influence of stack effect in inclined roadway. The results show that the mass velocity of smoke flowing into inclined roadway increases exponentially with the increase of vertical height of inclined roadway. For the problem of the disordered effect of exogenous fire on the mine ventilation and air flow system and the general law, due to the limited means of simulation, most scholars have limited their research on mine fires in alleyways where the fire point is located and the individual alleyways connected to it, or the part of the alleyway through which the smoke flow passes on the downwind side of the fire point for simulation analyses^11–17^. There are few studies on the law of smoke spread and airflow disorder in the fire period of the whole mine roadway network.

Hence, in this work, by applying the propagation law of the high-temperature smoke flow derived from similar duct experiments during the period of duct fires, a mine fire simulation study is conducted based on the TF1M3D computer simulation platform in the context of the entire mine field of Dayan mine. According to the simulation results, the corresponding measures are put forward.

## Experiments and simulation methods

### Experiments

#### Inclined single pipe test device

To obtain the mechanism of the wind flow turbulence caused by fire dynamics after the occurrence of exogenous fires in mines, the Froude similarity criterion was used to establish a duct experiment to simulate the basic form of the wind turbulence in the event of a fire in a real mine.

**Figure 1:**
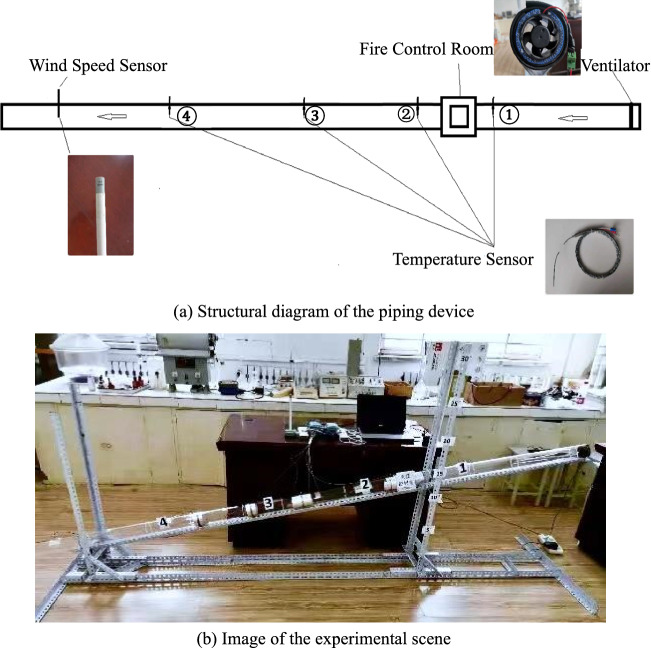
Single pipe test rig (device) for mine fire experiments.

The fire experiment alleyway part considers the heat exchange with the surrounding rock. The specific heat capacity of sand and gravel is 0.92 kJ (kg^−1^ °C^−1^), and the common specific heat capacity of steel and iron is 0.46 kJ (kg^−1^ °C^−1^). Since the experimental results have a high impact, a high-temperature-resistant transparent quartz glass tube with a specific heat capacity of 0.94 kJ (kg^−1^ °C^−1^) in the temperature range of 0–500 °C was used. The pipeline was 3 m long, Φ80 mm in diameter, with a fire control room in the middle. Standard solid alcohol was used as the fire source to simulate the uncontrolled combustion occurring in the single local tunnel. The pipeline was connected using a flexible and high-temperature-resistant silicone sealing joint, insulated with a high-temperature-resistant asbestos mesh sealing, and fixed with white steel hoops. The entire pipeline was placed on a sliding bracket with an adjustable angle, and temperature measurement points were set at certain distances at different locations near the fire source. The temperature at the center of the tunnel was tested using a thermocouple of the high-accuracy WRNK-191 type (accuracy class: I ± 0.75%t), and the temperature range was 0–800 °C. The wind speed is measured by W410C2 tubular miniature wind speed sensor with a measuring range of 0–5 m s^−1^ (accuracy class: ± 0.2 m s^−1^ + 3%of MV). In the experiment, a miniature powerful ventilation fan was used to supply air at the top of the press-in, and a duct-type wind speed sensor was installed with a range of 0–6 m s^−1^ at the outlet of the tunnel. Experimental data, such as the temperature and tunnel wind speed at the temperature measurement points, were collected through the Configuration King software, as shown in Fig. 1.

#### Loop system multiple pipe test device

**Figure 2:**
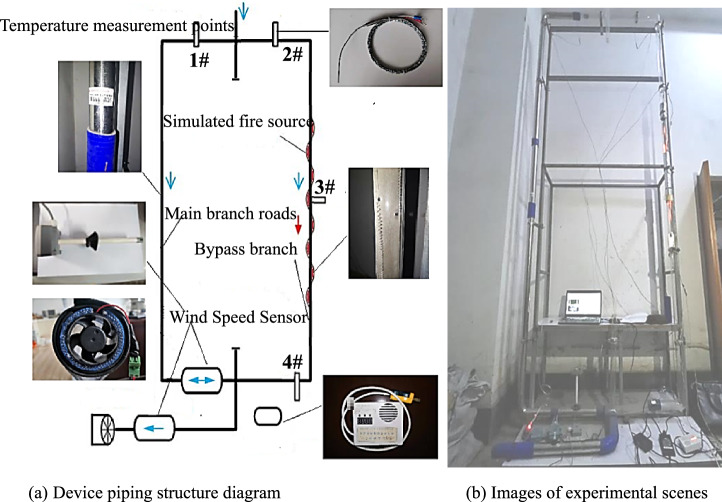
System piping test platform (device) for mine fire experiments.

Figure 2 shows the structure of the experimental model^18^, with an experimental pipe height of 3.8 m and a lifting support steel frame as the frame adjustable in the range of 2.2–4.8 m. In the experimental system, the main air path of the fire is a high-temperature resistant quartz tube with an inner diameter of Φ44 mm and an electronically controlled fire source. The side branch is a cooling metal tube with an inner diameter of Φ45 mm, the high-temperature pipeline is sealed with insulated and high-temperature resistant asbestos mesh and fixed with metal clips, and the rest of the pipeline is connected with good elasticity and high-temperature-resistant silica gel. The rest of the pipeline is connected using flexible high-temperature-resistant silicone joints. Adjustable tilt angle of the device, modification of complex connections such as expansion pipelines. The main air path can be arranged within the simulated fire source of the electric furnace wire, through the power regulator, to change the intensity of the fire source in real time to study the propagation of the high-temperature smoke flow under different fire source intensities. It can be pre-set to different configurations of the fire source for experimental requirements, automated control options, see Table 1 for details. Three temperature measurement points are set up in the main air path and one temperature measurement point in the side branch. The experiment uses the same equipment such as the temperature measurement thermocouple, wind speed sensor, fan and data acquisition platform as the single inclined roadway experiment. At the bottom end, two wind speed sensors are arranged, one in the main tunnel to measure the total wind speed of the experimental system and the other in the side branch. The experiments follow the same *Fr* similarity criterion in principle. When a fire occurs in the alleyway, the airflow in the alleyway is turbulent, and the smoke flow is mainly influenced by buoyancy.

**Table 1:** Experimental simulation of fire source voltage editor settings.

Fire burning time (s)	0	60	120	240	360	480	600	660	780
Analog fire control voltage (V)	0	180	220	220	180	160	110	80	0

### Ventilation system simulation software

The professional analysis software TF1M3D^19–22^ for the visualization and simulation of the ventilation system in the mine three-dimensional roadway network domain during the fire period can describe the development trend of the mine fire visually and dynamically, and realize the physical simulation of the disaster evolution process. TF1M3D is developed based on MATLAB and based on the theory of active wind network, which can realize the integrated calculation of normal ventilation and disaster process, and can describe the processes of unsteady ventilation, mine fire, coal and gas outburst, and reverse wind in mines. The software does not need to draw a ventilation network diagram to achieve a true description of the spatial structure of the entire mine roadway network. The three-dimensional image display in perspective can be used for multi-perspective observation by zooming, panning, and rotating. In the simulation calculation of mine fire, the convergence factor of wind flow stagnation and diversion with good convergence effect is introduced, and the thermal resistance change caused by the density change, as well as the gas accumulation and concentration overrun caused by the wind flow disturbance are considered. When describing the intensity of the fire source, consider the oxygen concentration of the wind flow and the problem of possible flashback. Through the TF1M3D platform, it is possible to scientifically predict the disaster and scope of coal mine fires, obtain disaster experience, evaluate the fire disaster resistance of mine ventilation systems, formulate scientific fire emergency plans, and provide decision-making basis for maximum disaster reduction and relief.

The experiments described above give a systematic account of the local smoke spread during the fire period. However, it represents the overall change in the mine when the fire occurs, and the local change is not sufficient to explain the overall mine network domain. Therefore, the TF1M3D computer simulation platform was used to simulate the smoke spread during the fire period for the entire Dayan mine^23^ to verify phenomena such as the backward flow of smoke from the main road into the side branch during the downstream ventilation fire. The parameters of the simulation platform were set in accordance with the propagation laws of the temperature and smoke flow during the fire period obtained from the experimental results^24^ and the real geographic environment of the coal mine. A 3D simulation model of the entire Dayan mine was thus established.

**Figure 3:**
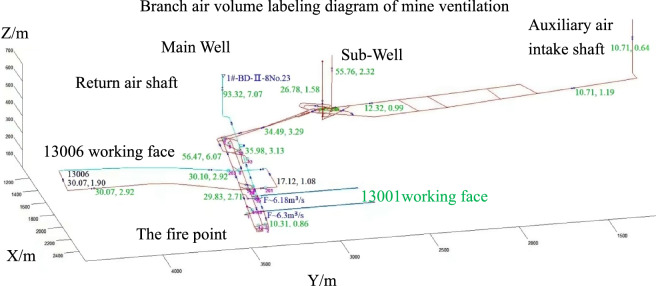
Three-dimensional wind volume annotation diagram of the TF1M3D simulation platform of Dayan Mine.

Figure 3 shows the 3D diagram of the wind volume labeling after modeling the Dayan mine. The mine adopts a ventilation arrangement with three inlets and one return. The total air volume is 93.32 m^3^ s^−1^, and the total wind pressure is 2233.02 Pa. The natural wind pressure is 166.153 Pa, the first number marked in green in the tunnel is the wind volume of the tunnel, the second number is the wind speed, and the purple color marks the number of dampers and regulating wind windows. The wind flow direction is indicated as follows: the branch with “○•” indicates the direction of wind flow, “•” is the forward direction of the wind flow. F is the local ventilation fan.

### Kinetic equations for the disaster process during mine fires

#### Calculation of the thermal resistance of ventilation during the fire period

During coal mine fires, thermal forces accompany the entire process of fire source combustion, and a force that hinders ventilation due to the fire source combustion occurs in the fire zone, which is the fire zone resistance. The fire zone resistance is an important factor in the formation of the throttling effect, and the strength of the fire source combustion affects the change in the fire zone resistance and also the throttling effect, and the three are consistent to some degree. The mathematical expression for the resistance in the fire zone is as follows:

1 $$ h_{t} = \frac{1}{2}c_{t} \rho_{2} u_{2}^{2} $$

where $\rho_{2}$ is the exit air density of the tunnel in relation to the fire source, kg m^−3^; $u_{2}$ is the exit velocity of the tunnel for the fire source, m s^−1^; $c_{t}$ is the resistance coefficient, which can be expressed as follows:

2 $$ c_{t} = \frac{2He}{{1 + He}} $$

where $He$ is the dimensionless heating criterion. The formula is as follows:

3 $$ He = \frac{s}{{c_{p} T_{0} }};s = \frac{Q}{mA} $$

where $s$ is unit mass of the high-temperature smoke flow in the fire zone to absorb the thermal energy, J kg^−1^; $T_{0}$ is stagnation temperature, K; $Q$ is heat generated due to combustion in unit time by the fire source within the fire zone, W; $c_{p}$ is the mass constant-pressure specific heat capacity of the flue gas, J (mol K)^−1^; $A$ is the cross-sectional area of the alleyway in which the fire source is located, m^2^; $m$ is the mass flow rate of the flue gas within the fire zone, kg (s m^2^)^−1^.

#### Basic equations for the movement-diffusion process of smoke flow in a shaft during a fire

During the period of mine fire, a large amount of high-temperature smoke flow is generated in the underground fire zone roadway. The flow of the high-temperature smoke flow in the underground roadway is given by any given branch j in the mine roadway network, and the convective diffusion equation for the roadway smoke flow is as follows:

4 $$ \frac{\partial c}{{\partial \tau }} + v\frac{\partial c}{{\partial x}} = E_{x} \frac{{\partial^{2} c}}{{\partial x^{2} }} $$

where $c$ is the average smoke concentration volume fraction at the tunnel section, %; $x$ is the location of the branch, m; $v$ is the average wind speed of the tunnel, m s^−1^; $E_{x}$ is the longitudinal mechanical dispersion coefficient of the wind flow, m^2^ s^−1^.

#### Calculation of fire wind pressure during the fire period

Once an exogenous fire occurs in the coal mine tunnel, the wind flow through the fire point is heated, and the thermal expansion of the wind flow is reduced because of the thermodynamic impact of fire in each branch of the tunnel, the existence of height differences will produce a slight pressure difference, i.e., the local fire wind pressure. The wind flow in the tunnel produces a continuous upward momentum in the ventilation network, and the fire wind pressure in any one circuit will be equal to the sum of the fire wind pressures of all the branches in the circuit.

The local (single branch) fire and wind pressure calculation formula is as follows:

5 $$ H_{F} {\text{ = h}}_{{\text{f}}} = \int_{{z_{1} }}^{{z_{2} }} {\Delta \rho gdz} $$

where $z$ is the height difference of the high-temperature smoke flow from position 1–2, m; $\Delta \rho$ is the difference in the air density in the tunnel before and after the fire, kg m^−3^.

The formula for calculating the fire air pressure of the system circuit:

6 $$ h_{F,b} = \sum\limits_{s = 1}^{{N_{s} }} {\left[ {P_{e,s} ,P_{e,s}^{(0)} } \right]} $$

where $b$ is the loop; s is the loop branch; $N_{s}$ is the number of loop branches; $p_{e}^{\left( 0 \right)} ,s$ is the differential pressure on $s$ before the fire, Pa; $h_{F,b}$ is the loop fire wind pressure, Pa.

## Results and discussion

### Experimental results and analysis of the Inclined single pipe test device

**Figure 4:**
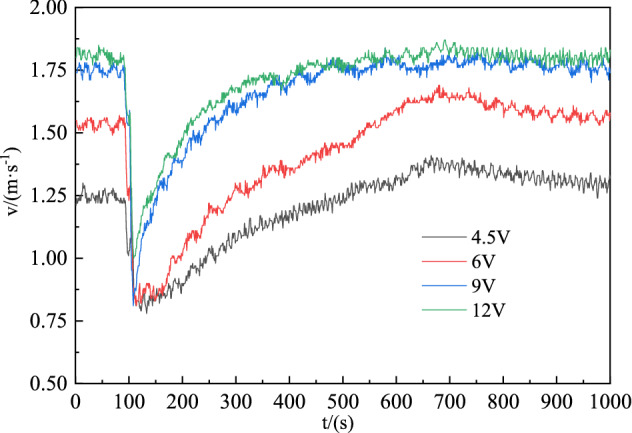
Variation in the wind turbulence at different air volumes.

The experiments simulated the change law of the high-temperature smoke flow with time after fire burning in the tunnel, and four ventilation capacities under ventilation fan voltages of 4.5, 6, 9, and 12 V were selected. As shown in Fig. 4, for the change in the air volume in the tunnel, the initial wind speeds are 1.2 and 1.9 m s^−1^, and the fire source starts to burn at 100 s. The air in the tunnel is heated and expands to a lower density, which produces a throttling effect to reduce the tunnel air volume and causes a disorder in the wind flow. The fire source burning intensity and ventilation power are positively correlated during the fire period. An increase in the ventilation power intensifies the burning of the fire source and produces more toxic and harmful gases. The stability of the ventilation in the tunnel gradually returns to the normal state as the burning intensity decreases.

**Figure 5:**
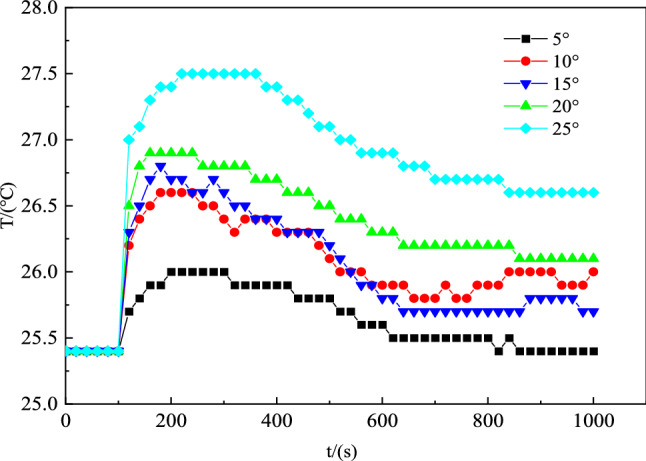
Temperature variation at measurement point 1 at different inclination angles.

As shown in Fig. 5, for the temperature change at the temperature measurement point 1 at a ventilator voltage of 4.5 V, the direction of the fire wind pressure generated by the combustion of the downstream ventilation fire source is opposite to the direction of the wind flow dynamics, as expressed by the fire wind pressure Eq. (5). With the increase in the inclination angle of the tunnel, the more evident the effect of fire wind pressure with the increase in the height difference of the tunnel, and the more significant the effect of the reverse retreat of the high-temperature smoke flow at temperature measurement point 1 with the increase in the inclination angle of the tunnel^25^.

### Experimental results and analysis of Loop system multiple pipe test device

Vertical system alleyway experiments are an extreme case of inclined alleyway fire simulation experiments. The following ventilator voltage settings are made: 4.5, 5, 6, 7.5 and 9 V. Table 2 presents the corresponding wind speeds in each line at the different voltages. In the five groups of experiments, only the main branch air flow reversed back when the ventilator voltage was 4.5 V, and the stability of the tunnel air flow increased with increasing air volume.

**Table 2:** Downstream ventilation fan voltage and corresponding wind speed.

Ventilator voltage level (V)	4.5	5	6	7.5	9
Total wind speed of main duct (m s^−1^)	1.69	1.79	1.91	2.2	2.62
Side-duct air velocity (m s^−1^)	0.26	0.34	0.45	0.65	0.78

**Figure 6:**
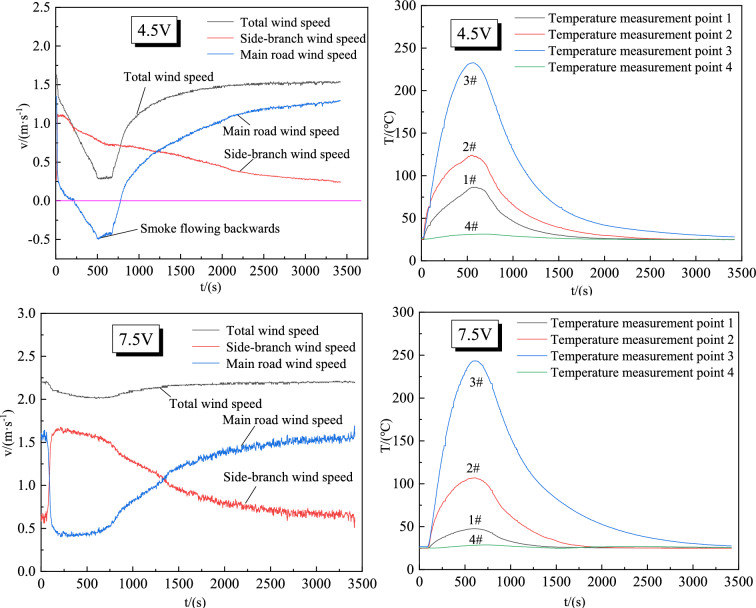
Experimental wind speed and temperature change.

Figure 6 shows the variation curves of the wind speed and the temperature at the corresponding measurement points in each lane at ventilator voltages of 4.5 V and 7.5 V. When the air flow is heated through the preset fire source, the gas is heated and expanded, thereby decreasing the air density to produce a density difference. The main air path produces a vertically upward fire wind pressure, which has an obstructing effect on downstream ventilation. Under the action of the fire wind pressure and fire zone resistance, the total air speed of the tunnel decreases, and with the increase in the ventilator capacity, the stronger the ability of the air flow to overcome the fire wind pressure and maintain ventilation stability. From the temperature data recorded at each measurement point, the temperature is found to be highest at the temperature measurement point 3, which is nearest to the fire source. The high-temperature smoke flow reaches upward to the temperature measurement point 2 under the action of the fire wind pressure and enters the side branch with the wind flow through the temperature measurement point 1.0A portion of the air flow is heated to flow through measurement point 4, causing a slight change in the temperature.

The fire source just appears, and the main road wind speed decreases, leading to a rapid increase in the side branch wind speed. The fire source intensity exhibits a rapid growth phase, the ventilation thermal resistance and fire wind pressure also increases, the pressure difference between the two ends of the main road increases, the main road wind speed decreases, and the wind speed in the side branch increases. The stronger the ventilation fan capacity, the more evident the change in the wind speed. The fire source intensity gradually reaches the peak, and growth tends to slow. The main road wind speed reaches the minimum value. When the ventilation capacity is low (4.5 V), the high-temperature smoke easily flows backwards. In the decay process of the fire source, the fire wind pressure and thermal resistance are reduced, the main air path pressure difference decreases, and the wind speed gradually returns to the value observed in the normal working period.

### Discrimination and analysis of critical wind speed due to turbulence

In the above experiment, the thermal resistance from the fire caused a change in the total airflow in the system. The fire wind pressure and thermal resistance from fires are the direct causes of wind flow disturbances in ventilation systems^26^. The wind volume shows a sharp variation because of the combined effect of throttling in the fire zone and the combustion of the fire the continuous burning of the fire source, due to the throttling effect of the fire area and fire source combustion, the air volume changed sharply. With the continuous combustion of the fire source, the thermal current heated through the fire area gradually spread, the fire pressure fully increased to the maximum, and a force opposite to the direction of the ventilation power was generated on the pipeline. Finally, under the combined action of the fire pressure and fire area resistance, the total air volume of the roadway reached the extreme value.

**Figure 7:**
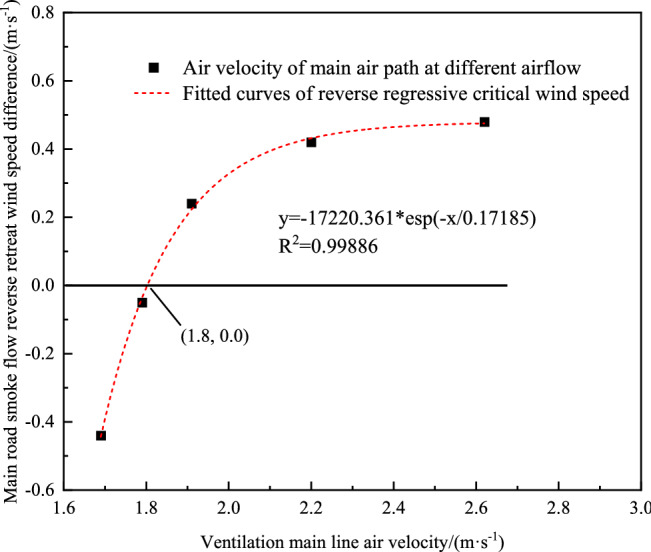
Determination of the critical wind speed for the turbulence in downward fire experiments.

The experimental results show that the fire wind pressure and ventilator power have critical values of equal magnitude and opposite direction in the downstream ventilation experiment, so that the downstream ventilation is in an equilibrium state through the nonlinear fitting of the wind flow retrogression at different wind speeds, as shown in Fig. 7, the critical wind speed for the downstream ventilation experiment is found to be approximately 1.8 m s^−1^. When the total air speed of the main control system is greater than 1.8 m s^−1^, the flue gas flow does not retrogress.

### Simulation results of fire in the Dayan mine

The choice of the mountainous section under the transporter (branch 205) as the fire starting point is in line with the real situation of the combustible location of the coal mine. The results show that at a ventilation fan speed of 540 r min^−1^, the weak flow area of the lower section of the roadway under the hauler can easily cause the fire main wind road smoke to flow backward, making the harmful gases to flow into the new air flow system in the mining area, into the working face of 13,006 to cause smoke flow infringement, which is a threat to the life and safety of workers in the working face.

**Figure 8:**
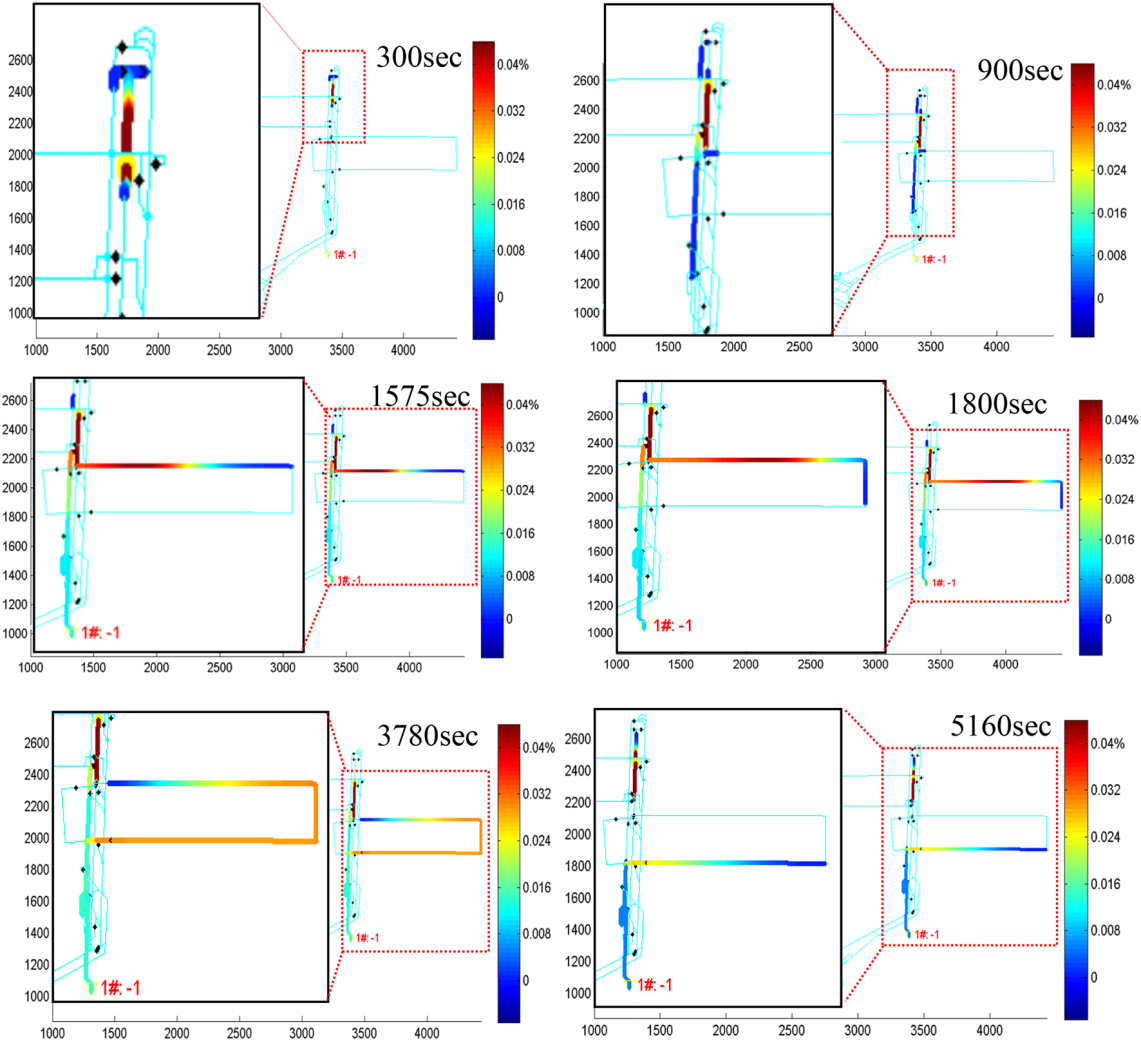
Process of CO gas spreading in the mine at the 13,006 working face due to downstream wind flow fire.

As shown in Fig. 8, the TF1M3D simulation platform^27^ shows a variation in the CO gas distribution of the flue gas in the mine network domain with time in the floor plan screen, where six screens are selected from several hundred screens. The fire starts at the preset fire point. Initially, the fire is not large, and the smoke stream drifts downstream with the wind to reach the bottom end of the downhill alleyway, passing through the dampers. As the fire continues to grow, the fire source burning intensifies, the fire wind pressure and thermal resistance increases, resulting in a gradual weakening of the wind in the downstream direction until the occurrence of wind flow stagnation, and then the smoke flow occurs in reverse retreat. The reverse retreating smoke flow enters from the central yard liaison road and firstly enters the return wind downhill through the dug-in return wind joint alley. Thereafter, a part of the high-temperature smoke flow enters the new air flow in the return mining alley of the 13,006 working face through the incoming wind joint alley, i.e. at the 900th s, it enters the working face from the wind alley entrance, and at the 1575 s, it reaches the 13,006 working face incoming from the wind entrance, which poses a hazard to the staff working at the comprehensive mining face. At 1800s, it flows out from the 13,006 working face, the fire slowly weakens with time, the fire wind pressure decreases, and the trend of smoke flowing backwards weakens. After the obstructive effect produced by the fire wind pressure was not sufficient to overcome the mine ventilation power, the air flow at the 13,006 working face began to gradually return to its original state before the fire, except for the transport downhill which continued to maintain reverse retreat. The smoke leaves the working face at 5160 s and enters the return air alley, passes through the dedicated return air downhill, reaches the return air shaft and discharges to the atmosphere outside the mine. The smoke flow diffusion results obtained from the simulation corroborate with the experimental high temperature smoke flow reversal phenomenon.

Through fire simulations, we fully understand the process of fire occurrence and the law of smoke flow spread as well as the change in the fire smoke flow trend in the mining area and its occurrence. This study explores the spreading law of mine fires and toxic CO gases in a complex network system underground, providing theoretical and technical support to scientifically formulate preventive planning measures, intelligently control disasters, and achieve disaster avoidance and mitigation.

### Fire emergency plan (measures) for lower mountain section

Ensuring the ventilation power of the ventilation system or increasing the ventilation capacity to maintain the reliability and stability of the ventilation system is conducive to overcome the fire intensity and quickly discharge the smoke flow. The TF1M3D simulation shows that the dangerous location for fires caused by smoke flow reversal is the weak flow area of the mine. The so-called “weak flow area” is on the non-main ventilation route of the mine, which is generally the non-mining and digging working face of the roadway wind supply area. The ventilation weak flow area formed in the downhill part of the downhill mining area below the working face of the Dayan Mine, where the ventilation power is weaker than the fire wind power. The following are some preplanned measures that can be taken to eliminate weak flow areas and prevent wind flow reversal.

1. Set aside a certain amount of spare air in advance in the coal mining working face or other roadways to apply the local regulation method in the mining area system. The coal mining working face should have the air volume within the specified range beyond the normal period. Once a fire occurs, to improve the ventilation strength of the weak flow area and prevent the downstream wind flow from backing up, the backup air volume should be activated and the regulating dampers can be used to change the leakage area and increase the air volume in the weak flow area.
2. Mine system (outside the mining area) air volume regulation. If there are difficulties in the internal regulation of the mining area and if regulation cannot be achieved, the air volume can be regulated from the range of the explored roadway, which is generally two-wing mining zoned ventilation, thereby increasing the wind resistance of one wing of the non-fire area to increase the air volume of that wing of the fire area and to ensure that the wing with reduced air volume is protected from the fire. The air volume of a wing of the fire area itself can be increased to ensure that the wind flow does not cause turbulence and naturally will not lead to a reduction in the air volume of that wing due to the smoke.
3. Another air volume adjustment method for the mine system (outside the mining area) is to adjust the main ventilation fan to increase the air volume. Changing the number of revolutions of the ventilator or employing a double-ventilator operation can help change the working characteristics and working points of the ventilator, thus improving the ventilation capacity of the ventilator and ensuring the stability of the ventilation system. This is one of the best methods that is not limited by the fire conditions of underground fires.
4. Accurate use of the anti-wind control disaster method. Unless there is a fire in the incoming air stream of the mine, it is generally not advisable to consider the entire mine backdraft. The underground ventilation form of Dayan mine adopts three inlets and one return, and the incoming air system has many passages and relatively complicated. Therefore, connections, so the entire mine backdraft is likely to cause a wide range of smoke stream pollution. Through the tunnel system to achieve local backdraft, open up the inflow flue, change the downstream wind flow fire to upstream wind flow fire, and prevent the backward flow of the smoke.

The realization of the above emergency wind control plan will not only ensure personnel safety, but also help achieve timely and improved efficiency of wind control. The necessity of intelligent ventilation (automatic adjustment and control of wind windows, and dampers) is particularly important to help control the wind flow in real time.

## Conclusions

1. In the downstream ventilation fire experiment, the ventilation dynamic direction of the main air path is opposite to the fire and wind pressure. With the increase of the intensity of the ignition source, the air volume decreases or even the phenomenon of smoke flow regression, which will not occur when the wind speed is greater than 1.8 m s^−1^.
2. Experiments and simulations mutually verify that when the wind flow fire is downward in the mine, the power of the fan is positively correlated with the ability of the main air path to maintain the original state, and the higher the power, the shorter the time it takes for the wind flow to return to the original state after the end of the fire.
3. The area with weak wind flow in the mine network area is the key area for preventing external fires. When the ventilator of Dayan Mine is less than 540 r min^−1^, the main air path may cause smoke flow reversal. The simulation of the plan is of great significance to the selection of disaster avoidance routes and the formulation of rescue plans during the mine fire period.

## Data Availability

The data that support the findings of this study are available within the article. All data generated or analysed during this study are included in this published article [https://doi.org/10.6084/m9.figshare.21340707.v1].
